# The Microscopic Anatomy of the Human Body, in Health and Disease

**Published:** 1846-10-01

**Authors:** 


The Microscopic Anatomy of the Human Body, in Health and Disease. Illustrated with numerous Drawings in colour. By *Arthur Hill Hassall*, Author of the British Freshwater Algæ, &c. London : Samuel Highley, 1846. Parts One and Two, 8vo.

The author informs us that " this work is to be completed in about twelve monthly parts, each comprising forty-four pages of letter-press and three carefully executed plates in colour ;" the price being 2*s.* 6*d.* each part. It is intended to embrace a systematic and copiously illustrated description of the various fluids and solids of the body, no structure or organ being omitted. A work of this nature, successfully completed, would at this particular time confer a real benefit on that large part of the profession, who are either engaged in the prosecution of minute anatomy or are interested in its progress. In the two parts that have already appeared, Mr. Hassall has considered the general characters of the lymph, the chyle, and the blood. As we shall have occasion in our next number to notice the valuable edition of Hewson's works, that has just been issued by the Sydenham Society, and in which the whole subject relating to these fluids has been ably discussed by the editor, Mr. Gulliver; and also the elaborate observations of Mr. Wharton Jones on the Development of the Blood-Corpuscle contained in the Philosophical Transactions for 1846, we can only devote a small portion of our space to that division of the work which is before us.

We are happy to pronounce on the whole a favourable opinion of " the Microscopic Anatomy." It contains a concise but comprehensive account of the subjects on which the author has hitherto treated. The opinions of the most eminent observers, English and Continental, are given with impartiality, and occasional references are made to the writings of the older microscopists, such as Malpighi, Leeuwenhoek, Della Torre, Hewson, and others ; the reader is thus put in possession of what is an indispensable requisite for arriving at the truth, the results, namely, of what has already been ascertained. Mr. Hassall does not, however, confine himself to anatomical details, but enters into such physiological questions as are more immediately connected with the ultimate structures; thus, after describing the blood-corpuscles, their uses are considered, and so with the lymph-globules, &c.

An opinion of the general style of the work may be formed by the author's remarks on the "Uses of the Red Corpuscles," in reference to Respiration.

"Observation has taught us the fact that the colour of the blood changes considerably, according as it is exposed to the influence of oxygen and carbonic acid gases, it becoming bright red under the influence of the former, and dark red, almost black, under that of the latter gas.

"Now the microscope has revealed to us the additional fact that the colouring matter of the blood resides within the red corpuscles; and hence we are led to infer that the changes of colour alluded to are accompanied by alterations in the condition of the colouring matter contained in those corpuscles.

"Further, the alterations of colour which have been mentioned take place not only in blood withdrawn from the system, but also in that which still circulates in the living body, the vital fluid being exposed in the lungs to the influence of the oxygen contained in the atmosphere, and to carbonic acid in the capillary system of vessels.

"But it is not merely a change of colour which the blood undergoes, or rather the coloured blood corpuscles undergo, on exposure to either of the gases particularised, but they also experience at the same time, as might easily be inferred, a positive change of condition, a portion of one or other of the gases to which the blood corpuscles are exposed being imbibed by them.

"That it is really the red corpuscles which absorb the oxygen, or the carbonic acid, as the case may be, admits of demonstration, and is proved by the fact that these gases lose but little volume when placed in contact with the *liquor sanguinis*, or serum of the blood.

"It is clear, then, that the coloured corpuscles are the seat in which these changes occur. Again, from the fact that the blood becomes bright red or arterial on exposure to oxygen, as in the lungs, and dark red or venous on being submitted to the action of carbonic acid, as in the capillaries, it has been inferred that they are first carriers of oxygen from the lungs to all parts of the system, and second, vehicles for the conveyance of carbon back again to the lungs.

"This inference is correct as far as it goes, but it fails to explain why the imbibition of oxygen or carbonic acid gases should be accompanied by changes in the colour of the blood; and it also fails to show why those gases themselves should be imbibed." P. 36.

The author, after giving Liebig's theory as to the manner in which oxygen acts upon the protoxide of iron existing in the corpuscles of the venous blood, thus proceeds:

"Venous blood, then, exposed to the air gives out carbonic acid and absorbs oxygen, but arterial blood submitted to the same influence gives out oxygen, and acquires carbonic acid, the seat of these changes being the red corpuscles.

"It will be seen on reflection, that, according to the views just propounded, the surplus amount of oxygen which exists in the peroxide becomes disengaged in the reduction of that oxide to the state of protoxide: during circulation in the capillaries, this surplus is chiefly expended in the elaboration of the different secretions which are continually being formed in the various organs of the body. Such is the *corpuscular theory of respiration.*" P. 37.

The plates representing the various classes of corpuscles found in the blood, are characteristic, though the roughness of lithography is ill adapted for the more minute objects depicted, such as the rouleaux or piles of the red particles of man (Plate 1, fig. 4). The figure of the large discs of the Siren Lacertina, is excellent. The segment to illustrate the small arteries and veins of the frog's foot, with the intervening capillaries, parts it may be remarked en passant, which often puzzle the young microscopist to distinguish, might have been more happily chosen. We must also observe that the account which Mr. Hassall has given of the circulation in the chick is not very clear, the details appearing to us, if we rightly apprehend the description, to correspond rather to the umbilical vesicle and its blood-vessels (omphalo-mesenteric) than to the allantois with the umbilical arteries and veins. Notwithstanding these defects, the work of Mr. Hassall may be regarded as an useful guide to those who wish to become acquainted with the latest views upon microscopic anatomy; and on these grounds we have pleasure in recommending it to the notice of our readers.

---

